# Myasthenia gravis: understanding treatment patterns and direct medical costs in the Czech Republic

**DOI:** 10.1186/s13023-024-03504-3

**Published:** 2024-12-20

**Authors:** Gleb Donin, Karla Mothejlová, Magda Horáková, Stanislav Vohanka

**Affiliations:** 1 Department of Biomedical Technology, Czech Technical University in Prague, Kladno, Czech Republic; 2 Department of Neurology, ERN EURO-NMD Center, University Hospital Brno, Brno, Czech Republic; 3 Faculty of Medicine, Masaryk University, Brno, Czech Republic

**Keywords:** Myasthenia gravis, Treatment patterns, Direct healthcare costs

## Abstract

**Background:**

Myasthenia gravis (MG) is a rare autoimmune disorder with significant clinical implications, including life-threatening myasthenic crises and exacerbations. Understanding real-world treatment patterns, especially associated direct medical costs, is essential for the effective management of healthcare delivery.

**Methods:**

We conducted a descriptive cohort study using health administrative claims data from the Czech Republic covering more than 1,500 prevalent MG patients. Data were analysed for healthcare resource utilization, medication costs, and hospitalization rates related to MG and its complications.

**Results:**

Acetylcholine inhibitors and corticosteroids were widely prescribed, with 91.1% and 75.2% of patients receiving them at least once, respectively. Immunosuppressive therapy was given to 45.2% of patients. Myasthenic crises occurred in 2% of patients, with a mean hospitalization cost of 21,020 EUR, while exacerbations occurred in 9.2% of patients, with lower costs (5,951 EUR per hospitalization). Outpatient intravenous immunoglobulin and plasma exchange therapies incurred additional costs of 20,700 EUR and 18,206 EUR per person-year, respectively. The mean total cost per patient-year was 1,271 EUR, with significant cost differences among patients with different treatment patterns.

**Conclusion:**

This study offers real-world insights into the treatment patterns and associated direct medical costs of MG in the Czech Republic. Myasthenic crises and exacerbations pose considerable cost burdens, while outpatient therapies and common pharmacotherapies are less costly. These findings are vital for healthcare planning, economic evaluation, and resource allocation, potentially leading to enhanced patient care and outcomes.

**Supplementary Information:**

The online version contains supplementary material available at 10.1186/s13023-024-03504-3.

## Introduction

Myasthenia gravis (MG) is a rare autoimmune disorder where autoantibodies attack neuromuscular junction proteins, impairing neuromuscular transmission. This chronic condition leads to various symptoms, such as diplopia, ptosis, and muscle weakness, affecting functions such as breathing and swallowing [1]. Severe cases may result in myasthenic exacerbations (ME) and even life-threatening myasthenic crisis (MC), characterized by respiratory muscle paralysis [2, 3]. MG significantly impacts patients’ quality of life, posing both medical and societal burdens [4]. Despite its rarity, understanding MG’s complexities is crucial for effective management and intervention selection.

In Europe, the estimated prevalence of MG varies, ranging from 2.6 per 100,000 in Hungary in 2015 to 36.1 per 100,000 in Sweden in 2020 [5]. The incidence rates have a bimodal distribution in women, peaking around the ages of 30 and 50 and steadily increasing in men with age [6]. Review studies have shown a substantial increase in prevalence in recent years [5, 7–10]. The increasing prevalence of MG could be attributed to factors such as an aging population, reduced disease-related mortality due to improved treatments, and enhanced recognition of the condition [11].

Acetylcholinesterase inhibitors (AChE-I) are the primary symptomatic treatment, but many patients also require long-term immunosuppressive therapies such as corticosteroids, nonsteroidal immunosuppressants (IST), intravenous immunoglobulin (IVIG), or plasma exchange (PLEX) [1, 3]. PLEX and IVIG are expensive but effective for managing myasthenic exacerbations and crises. Emerging treatment options such as monoclonal antibodies (biologicals) add complexity to MG management, raising questions about their cost compared to traditional therapies such as PLEX and IVIG [1].

Analysing the cost of illness and the socioeconomic burden of a MG serves as a valuable public policy tool, aiding in the prioritization and justification of healthcare and prevention policies. The authors note the absence of any national study comprehensively analysing resource use across all healthcare sectors. Additionally, there is a lack of similar studies in the Central European region (CEE). Several non-CEE studies have investigated the social and economic burden of MG [1, 2, 4–6]. Further investigations are necessary to provide reliable data on costs and resource utilization for individual patients with MG.

This paper aims to provide an estimation of health resource utilization, specifically focusing on (1) the direct costs associated with the treatment of MG and MC in the Czech Republic and (2) the distribution of these costs among inpatient, outpatient, and pharmaceutical services.

## Methods

### Study design and data sources

We performed a descriptive cohort study using a health administrative claims (HAC) database in the Czech Republic. This study aimed to describe health care utilization and calculate associated costs in a cohort of patients with prevalent MG.

The HAC database contains more than 550 million claims, including medication prescriptions for more than 4.5 million unique patients insured by six (from a total of seven) public health insurance funds in the Czech Republic. This equalled 44% of the Czech population as of December 2021 and provided a geographically well-diversified sample. Health insurance is compulsory in the Czech Republic, and citizens as well as permanent residents are insured by public health insurance funds; hence, these sources of data can be considered representative of national health care as well as of MG management, as the majority of its costs are claimed through these funds. The data were available from January 1, 2017, to December 31, 2021 (the *study period*).

The HAC data used were deidentified; therefore, the approval of the internal review board was not required for this descriptive study. Additional HAC database details are presented in the supplementary material.

### Study cohort

To define the prevalent cohort, individuals with longitudinal HAC records were included if they (i) had a diagnosis of MG reported in the selection period from January 2017 to December 2020, (ii) had at least one inpatient or two outpatient records with MG diagnosis, whereas the time difference between them was at least 30 days, and (iii) had a MG-related therapy initiated (hospitalization with MG as the primary diagnosis, AChE-I, IST, IVIg, or PLEX; see details below) within the selection period. We did not include patients diagnosed in 2021 to ensure at least one year of follow-up. The cohort entry date, i.e., the *index date*, was defined as the date of the first reported MG. We excluded patients younger than 18 years as of the *index date*.

### Analysed outcomes

Patient age and sex were obtained from the HAC data at the index date. MG diagnosis was defined using the ICD-10 code G70.0. The AChE-I, corticosteroids (CS) and IST medications used were identified using ATC codes (see supplemental Table S1). Outpatient neurology visits were identified using claims with a specialty code for neurology and a primary diagnosis of G70.0. IVIg administration was defined using ATC code J06BA02, and PLEX was defined based on specific procedure codes (detailed in supplemental Table S1).

In this study, based on the HAC data, we established the MC and ME as follows: a MC was identified as a hospitalization with a primary diagnosis of MG, length of stay over 2 days, IVIg or PLEX treatment, and reported ventilation procedures (codes detailed in supplemental Table S1). ME was identified similarly but without ventilation procedures. Our operational definitions for ME and MC were similar to that of previous studies [12–15].

This study measured outcomes, including resource utilization and costs of MG-related medications, neurologist visits, IVIg and PLEX therapies, and costs associated with treating MCs and MEs. Assessment was conducted during the follow-up period, extending from the index date until patient death or the last recorded claim within the study period, whichever occurred first.

Medication costs in local currency (Czech crown – CZK) were directly extracted from HAC databases. The duration of pharmacotherapy was defined using the World Health Organization’s Defined Daily Dose. For medications (CS, AChE-I, and IST), we evaluated total package numbers and mean costs per person-year (PY) of pharmacotherapy.

IVIg administration and PLEX procedures in outpatient settings were quantified in therapy cycles. A cycle was defined as a continuous sequence of IVIg or PLEX claims with delays not exceeding seven days. IVIg cost analysis included medication costs and administration costs (procedure codes are detailed in Supplemental Table S1). We considered all claims submitted on the same day by an outpatient healthcare provider who conducted a PLEX procedure for a patient as outpatient PLEX costs.

The costs of MC and ME were calculated as the sum of costs for medications and procedures. IVIg and PLEX therapies were evaluated as the mean cycle costs and mean costs per PY of therapy. For hospitalizations (MCs and MEs), we estimated the number of hospitalizations per 100 PY using the patients’ follow-up periods, mean length of stay and mean single hospitalization costs.

During the follow-up period, direct healthcare costs for MG management were estimated, including healthcare resource utilization (outpatient therapy, specialist visits, and inpatient care) and medication costs. Costs are presented as the mean values in Euros (EUR) using the annual mean currency exchange rate set by the Czech National Bank for 2021 (25.645 CZK for 1 EUR). Further details on the HAC database, medication approach, IVIG and PLEX treatment estimation, and prevalence estimation methodology are provided in the supplementary material.

The data processing and analysis were conducted using R for Windows.

## Results

### Participants

Of the 3,206 patients who were initially identified with MG diagnosis claims, 1,523 met the study’s inclusion criteria. The prevalent cohort comprised 53% women, with a median age of 67 years (Table 1). Over the follow-up period (2017–2021), 16% of patients died. The median follow-up duration was 1,418 days.

**Table 1:** Patient characteristics

Characteristic	*N* = 1 523
**Sex**	
Female	809 (53%)
Male	714 (47%)
**Age**	
Median (IQR)	67 (53, 75)
Mean (SD)	63 (16)
**Follow-up period**,** days**	
Median (IQR)	1 418 (920, 1 723)
Mean (SD)	1 281 (489)

Note: IQR = Interquartile range; SD = Standard deviation

Table 2 shows the resource use and medication costs for MG treatment. AChE-I were the most common medication category, prescribed to 91.1% of patients, with an average annual cost of 305 EUR per patient. Pyridostigmine was the most commonly prescribed AChE-I (89%), followed by Ambenonium (13%) and Distigmine (10%). Neostigmine (7%) was the least commonly used agent. Three-quarters (75.2%) of patients received CS, with Prednisone being the most common (78%), followed by Methylprednisolone (40%). The average annual cost of corticosteroid therapy was 50 EUR per patient. IST was prescribed to 45.2% of patients, with a mean annual cost of 842 EUR. Azathioprine was the most commonly used IST (80%), followed by Mycophenolate (13%), Cyclosporin (8%), Methotrexate (5%), and Tacrolimus (2%).

**Table 2:** Outpatient medications during the follow-up period

Medication	*N*	%	Packages (*n*)	Mean package count PY/therapy	Mean cost per PY/therapy, EUR
AChE-I	1 388	91.1	35 675	13	263
CS	1 145	75.2	43 564	23	44
IST	688	45.2	15 379	17	414

Note: AChE-I = acetylcholine inhibitors; CS = corticosteroids; IST = Immunosuppressive therapy; PY = person year

Additional MG-related outpatient therapies are presented in Table 3. Only 7 patients (0.5%) received biological therapy during the study, totalling 33,336 EUR. IVIg therapy was administered to 58 patients (3.8%) at an average cost of 20,700 EUR per PY, with a mean cycle cost of 1,700 EUR. PLEX was conducted in 62 patients (4.1%), costing 18,206 EUR per PY of therapy, with a mean cycle cost of 1,380 EUR.

**Table 3:** Outpatient IVIG and PLEX treatments during the follow-up period

Therapy	%	Cycles (*n*)	Cycles per PY	Mean cycle costs, EUR	Mean cost per PY/therapy, EUR
IVIg	3.8	977	12	1 700	20 700
PLEX	4.1	506	14	1 380	18 206

Note: IVIg = intravenous immune globulin therapy; PLEX = plasmapheresis; PY = person-year.

In total, 1,407 patients (92.4% of the total study cohort) visited a neurologist, with an average of 3.2 visits per year. The average visit cost was 22 EUR. Consequently, the average annual cost per patient for visits to a neurologist was 71 EUR.

Table 4 summarizes the occurrences of MCs and MEs in the prevalent cohort during the follow-up, along with the associated healthcare costs. During the follow-up, 140 individuals (9.2%) experienced ME, while only 30 patients (2%) experienced MC. ME resulted in 232 hospitalizations, while MC led to 35 hospitalizations. The hospitalization rate per 100 person-years was 4.3 for MEs and 0.6 for MCs. Significant differences were observed in costs and mean length of stay (LOS) between MEs and MCs. The average cost per hospitalization for MCs was 21,020 EUR, with a mean LOS of 32.6 days, compared to 5,951 EUR per hospitalization for MEs, with a mean LOS of 11 days. Additionally, there were 470 MG-related hospitalizations for reasons other than ME and MC, affecting 311 patients (20.4%) in the prevalent cohort, with an average cost of 1,560 EUR per hospitalization and a mean LOS of 8.6 days.

**Table 4:** MG-related hospitalizations during the follow-up period

Hospitalization	*N*	*N* per 100 PY	LOS, mean, days	Mean single hospitalization costs, EUR
Myasthenic exacerbation	232	4.3	11.0	5 951
Myasthenic crises	34	0.6	32.6	21 020

Note: PY = person-year; LOS = length of stay

20% of ME hospitalizations were associated with admission to the intensive care unit (ICU). Patients with MG-related hospitalizations, including those in the ICU, had a mean LOS of 14.2 days, compared to 10 days in patients without an ICU stay. The mean ICU LOS was 6.4 days. The average cost of a single hospitalization with the ICU was 7,964 EUR, compared to 5,324 EUR without the ICU.

The mean total cost per PY was 1,271 EUR, which increased to 13,368 EUR for the top 5% of expensive patients with follow-up periods exceeding 1 year. The estimated annual direct medical cost of MG in the Czech Republic was 4.4 million EUR.

## Discussion

Our findings revealed widespread use of AChE-I (91.1%) and CS (75.2%) among prevalent patients. Prednisone was the most commonly prescribed corticosteroid, received by 78% of patients, followed by Methylprednisolone (40%). In contrast to a study from Italy [10], our research found a higher percentage (45.2%) of patients received IST, primarily Azathioprine (81%). Overall, IST, CS, and AChE-I were relatively affordable, costing less than 450 EUR per person-year of therapy.

Biological therapy, specifically Rituximab, was administered to a limited number of patients, with only seven individuals (0.5%) receiving this treatment at a total cost of 33,336 EUR. In Germany, Rituximab was prescribed to 6% of patients, while a US study reported that 7% of patients were treated with Rituximab [16, 17]. These variations highlight differences in treatment approaches across different regions. In contrast, outpatient IVIg and PLEX therapies (including immunoadsorption) were more common in our study, with 58 (3.8%) and 62 (4.1%) patients, respectively.

Outpatient IVIg treatment averaged 16 cycles per patient, costing approximately 1,700 EUR per cycle and totalling 20,700 EUR per PY of IVIg therapy. PLEX therapy involved an average of 8 cycles, with each cycle costing approximately 1,380 EUR, resulting in mean costs per PY of PLEX therapy of 18,206 EUR. Our study notably found IVIg treatment to be more costly than PLEX, contrary to findings from a US study [3]. However, an Indian study supported our analysis, confirming IVIg as the more expensive option, possibly due to variations in labour and medication costs among countries [18].

In the prevalent MG cohort, 140 patients (9.2%) experienced ME, while 30 patients (2%) experienced MC. This results in an estimated annual rate of approximately 5% for MC/ME. A German study [19] reported a fluctuation in MC/ME occurrence between 4.5% and 5.8% from 2017 to 2020. Our study observed a mean length of stay of 11 days for MEs and 32.5 days for MCs, contrasting with the mean length of stay of 8.58 days for MEs reported in a US study [20]. The average treatment cost for MCs was approximately 21,020 EUR, which was significantly greater than the cost for MEs, which was approximately 5,951 EUR per hospitalization. The pattern of higher inpatient costs for MCs is supported by several studies [3, 18, 21].

The size of the prevalent cohort leads to a crude MG prevalence estimate of approximately 28.2 per 100,000 inhabitants as of 31st December 2021. Our study included 53% women, in contrast to a study from 2022 where 53% were male [13]. However, a US study showed 56% females [17]. This result highlights the variability in sex distribution across different geographic sectors. The unpublished analysis of the patient registry for MG in the Czech Republic provided a similar prevalence value of 27 per 100,000 persons. This finding supports the approach used for the MG cohort definition in this study and confirms the findings of other research indicating increasing prevalence [5, 7–10].

The analysis also examined the use of the ICU in patients with ME, excluding MC. A study that focused on the analysis of the LOS and treatment of MG patients reported that patients in the ICU had an average length of stay of 16 days, compared to 14.2 days in our study and 8 days in non-ICU hospitalization, compared to 10 days [20]. The mean single hospitalization costs with the ICU were significantly greater at 7,964 EUR, compared to 5,324 EUR without the use of the ICU, supporting the results of an Indian study analysing direct and indirect costs [18].

Our study has several limitations. First, because the data originated from administrative claims and discharge records, it is possible that not all relevant information was recorded. Second, it is essential to note that this analysis is descriptive rather than causal. Despite these limitations, the study offers valuable insights and data for various healthcare sectors.

The study dataset includes data from all regions of the country and six out of the seven health insurance funds. In the Czech Republic, public health insurance is mandatory, and individuals can freely choose from one of the seven health insurance funds. While there may be slight variations in regional coverage and age distribution across the funds, treatment protocols and reimbursement policies are standardized nationwide. Therefore, the dataset is expected to be representative of the broader population in terms of diagnoses and treatments.

The study results are originated solely from the Czech Republic. Other Central European countries (especially Slovakia) are showed to have similar healthcare financing structures [22] and face comparable public health challenges [23]. For example, Slovakia and Poland had the MG prevalence estimates similar to Czech, 24.5 and 22.6 respectively [24, 25]. Variations in healthcare policies, economic conditions, or demographic factors could affect the generalizability of the results of this study, while the core findings are likely to remain relevant.

In addition to direct medical costs, the burden of MG extends to several other areas, including indirect costs, direct non-medical costs. MG impacts patients’ productivity by causing muscle weakness and fatigue, which can lead to frequent absences from work, reduced work hours, and decreased overall efficiency in daily tasks. Productivity losses have been shown to account for up to 40% of total all-cause costs for employed MG patients [26]. Informal care can account for up to 10% of direct medical costs [27]. Additionally, direct non-medical costs, including transportation, home modifications, and other out-of-pocket expenses, should be considered to capture the full financial impact on patients and their families [28]. Moreover, incorporating patient-reported outcomes in future research could offer valuable insights into the quality of life among and treatment satisfaction among MG patients [16]. This can help identify unmet needs and guide the development of patient-centered care strategies.

## Conclusion

This study provides an overview of MG-related direct health care costs for patients in the Czech Republic. Myasthenia crises and exacerbations could be considered the most significant components of MG-related direct costs. IVIg and PLEX are also relatively cost-intensive therapies. Common MG pharmacotherapies (IST, CS, AChE-I) and outpatient visits were the least expensive. Future studies are needed to provide a more comprehensive understanding of the socio-economic burden of MG.

## Electronic supplementary material

Below is the link to the electronic supplementary material.

Supplementary Material 1

## Data Availability

The data remain property of the insurance companies and cannot be shared without their permission.

## Abbreviations

AChE-I: Acetylcholinesterase inhibitors
ATC: Anatomical therapeutic chemical
CEE: Central European region
CS: Costicosteroids
CZK: Czech crown
EUR: Euro
HAC: Health administrative claims
ICD-10: International Classification of Diseases
ICU: Intensive care unit
IST: Immunosuppressants
IVIg: Intravenous immunoglobulin
LOS: Length of stay
MC: Myasthenic crisis
ME: Myasthenic exacerbation
MG: Myasthenia gravis
PLEX: Plasma exchange
PY: Person-year
