# Supplementary material for: Myasthenia gravis: understanding treatment patterns and direct medical costs in the Czech Republic
Source: Orphanet J Rare Dis. 2024 Dec 20;19:472. doi: 10.1186/s13023-024-03504-3 (PMC11662583; doi:10.1186/s13023-024-03504-3)
Supplement: Supplementary file 1 — Supplementary Material 1 [file 13023_2024_3504_MOESM1_ESM.docx]

# Supplementary materials

**Technical details**

The HAC database includes the following data: age and sex, deidentified medical (inpatient and outpatient), drug and device claims data. International Classification of Diseases, Tenth Revision (ICD-10); Czech list of medical procedures codes; and site of service codes were used in medical claims. Medication administration and prescription claims used the national drug code from the list of reimbursed medicinal products (issued by State Institute for Drug Control); the list includes drug name, dosage form, drug strength, the code of Anatomical Therapeutic Chemical (ATC) Classification System and other drug-related data. Device claims utilize the local lists of reimbursed medical devices in outpatient and inpatients settings. For inpatient care the database also contains information on in-hospital death, length of stay, and intensive care units (ICU) days.

Individuals not continuously insured in a single public health insurance fund after the *index date* were excluded from the analysis in order to avoid misclassifications and loss to follow-up.

The ICD diagnoses in administrative data, especially in outpatient settings, may not always accurately reflect the presence of a disease. This can happen because diagnoses are sometimes based on incomplete or uncertain information, such as initial screenings or patient reports. As a result, the assigned codes may not fully match the actual condition. To address this issue, we applied a widely used approach that requires at least two outpatient records with an ICD-10 code for MG [1–3].

A medication treatment period was constructed for every patient and for each medication group. The medication treatment period for medication group was defined as the sum of not- overlapping time intervals representing the amount of Defined Daily Doses (DDD) in the claimed medication package. We extended DDD-based intervals by a factor of 25% in order to address different levels of adherence in patients.

Outpatient IVIg and PLEX treatment were analyzed in therapy cycles (the sequence of IVIg / PLEX without delays longer than one week). We considered patients “on IVIG” any time within 30 days of any IVIg administration. The same approach was applied to PLEX treatment. The IVIG / PLEX treatment period was defined as the sum of not- overlapping time “on IVIG” / “on PLEX” intervals.

The costs for inpatient and outpatient procedures were estimated by the number of points claimed to health insurance funds, and then converted to Czech crowns by rate 1 point = 1 CZK.

The prevalence of MG was determined per 100,000 persons by extrapolating the population insured by six (out of seven) health insurance fonds up to whole population in the Czech Republic. The incidence was calculated for 100,000 PY calculated across all *the incident cohort*.

**Table S1 Variables definitions**

| **Variable** | **Role** | **Definition** |
| --- | --- | --- |
| **Medications** |  |  |
| Acetylcholinesterase inhibitors (AChE-I) | Exposure and outcome | ATC code N07AA |
| Immunosuppressant therapies (IST) | Exposure and outcome | ATC codes L04AX01, L04AD01, L04AA06, L04AX03, L04AD02 |
| Corticosteroids (CS) | Outcome | ATC codes H02AB07, H02AB04, S02BA06, H02AB08 |
| Neurologist visit | Outcome | Specialty code 209  Primary diagnosis is ICD-10 G70.0 |
| Intravenous immunoglobulin (IVIg) | Exposure and outcome | ATC code J06BA02  Procedures: 09220, 09223, 06115, 09227 |
| Plasmapheresis (PLEX) | Exposure and outcome | Procedures: 22363, 18580, 22371, 22373 |
| Myasthenic crisis (MC) | Outcome | Primary diagnosis is ICD-10 G70.0  Length of stay 3+ days  IVIg and/or PLEX during hospitalization  respiratory support, intubation, or mechanical ventilation during hospitalization: procedure codes 90901, 90902, 90903, 90904, 90905, 90906, 90907, 90889, 90890, 91926 |
| Myasthenic exacerbation (ME) | Outcome | Primary diagnosis is ICD-10 G70.0  Length of stay 3+ days  IVIg and/or PLEX during hospitalization  Without respiratory support, intubation, or mechanical ventilation during hospitalization |

**Analysis of incident cohort**

We also performed sub-group analysis on incident MG patients in order to analyze temporal differences in hospitalization. The *incident MG cohort* were selected from the *prevalent MG cohort* according to the following criteria: (i) first inpatient or outpatient record with an MG diagnosis (*index date*) from January 2018 till December 2020; (ii) patients with available claims data (at least one inpatient/outpatient or drug prescription record) in calendar year prior to the year of the *index date*. For *incident MG cohort* we identified initial MC and ME hospitalizations (the closest to index date) and following (any other during the *follow-up period*).

The following table presents an additional analysis of the direct healthcare costs associated with hospitalizations for *incident MG cohort*. The hospitalizations were stratified as "initial" and "following" based on their order in the patient history. The most significant in terms of cost was the initial hospitalization due to MC, with 10 occurrences and a mean single hospitalization cost of 26,479 EUR. There were 11 following hospitalizations due to MC with a mean single cost of 18,663 EUR. There was no significant difference in the mean single hospitalization costs for incident patients presenting Myasthenia exacerbation. For the 31 initial hospitalizations, the costs were equal to 6,855 EUR, and for the 47 subsequent hospitalizations, it was 6,961 EUR.

**Table S2 Hospitalization costs for the incident cohort**

| **Type** | **Initial** | **Following** | **Mean single initial hospitalization costs, EUR, 95% CI** | **Mean single following hospitalization costs, EUR, 95% CI** |
| --- | --- | --- | --- | --- |
| Myasthenia  exacerbation (ME) | 31 | 47 | 6 855  (5 869, 7 842) | 6 951  (5635, 8267) |
| Myasthenia crisis  (MC) | 10 | 11 | 26 479  (16 774, 36 184) | 18 663  (13987, 23339) |

**References**

1. Murai H, Hasebe M, Murata T, Utsugisawa K. Clinical burden and healthcare resource utilization associated with myasthenia gravis: Assessments from a Japanese claims database. Clinical and Experimental Neuroimmunology. 2019;10:61–8.

2. Mevius A, Jöres L, Biskup J, Heidbrede T, Mahic M, Wilke T, et al. Epidemiology and treatment of myasthenia gravis: a retrospective study using a large insurance claims dataset in Germany. Neuromuscular Disorders. 2023;33:324–33.

3. Wartmann H, Hoffmann S, Ruck T, Nelke C, Deiters B, Volmer T. Incidence, Prevalence, Hospitalization Rates, and Treatment Patterns in Myasthenia Gravis: A 10-Year Real-World Data Analysis of German Claims Data. Neuroepidemiology. 2023;57:121–8.
